# Impact of ultrasound angiography combined with fine needle aspiration for the diagnosis of thyroid nodules

**DOI:** 10.1097/MD.0000000000017286

**Published:** 2019-09-27

**Authors:** Jing Wang, Xiao-Gang Bai, Zhe Liu

**Affiliations:** a Department of Endocrine and Metabolism; b Department of Cardiology, Yan’an University Affiliated Hospital, Yan’an, China.

**Keywords:** fine needle aspiration, sensitivity, specificity, thyroid nodule, ultrasound angiography

## Abstract

**Background:**

This study aims to systematically investigate the impact of ultrasound angiography (UA) combined with fine needle aspiration (FNA) for the diagnosis of thyroid nodules (TNs).

**Methods:**

The following electronic databases will be searched: MEDLINE, EMBASE, Cochrane Library, PsycINFO, Web of Science, Cumulative Index to Nursing and Allied Health Literature, Allied and Complementary Medicine Database, Chinese Biomedical Literature Database, and China National Knowledge Infrastructure. We will search them from their inceptions to the present without language limitations. We will consider all case–controlled studies on investigating the impact of diagnosis UA combined FNA for TNs. We will apply Quality Assessment of Diagnostic Accuracy Studies tool to assess methodological quality for all eligible studies.

**Results:**

In this study, outcomes consist of sensitivity, specificity, positive likelihood ratio, negative likelihood ratio, and diagnostic odds ratio. All these outcomes will be analyzed to evaluate the diagnostic accuracy of UA combined with FNA for TNs.

**Conclusion:**

This study will provide evidence of the diagnostic accuracy of UA combined with FNA for TNs.

**Systematic review registration:**

PROSPERO CRD42019138884.

## 1 Introduction

Thyroid nodules (TNs) are very common health problems in clinical practice among the adult population.^[1–3]^ They are commonly diagnosed as noncancerous or cancerous.^[4,5]^ It has been reported that the prevalence of TNs is about 19% to 68% of the healthy population,^[6]^ and it is higher in women than men with tends of increased age. Specifically, TNs are commonly clinical findings of 1% to 5% at physical examination, and 20% to 70% at ultrasound examination.^[7,8]^ Thus, it is very important to detect TNs early and to lead a better prognosis.^[9–14]^

Ultrasound angiography (UA) has been widely used as the imaging modality choice for early diagnosis due to its wide availability.^[15,16]^ In addition, fine needle aspiration (FNA) is also widely utilized as one of the most common diagnosis option for TNs.^[17–20]^ However, the single diagnostic accuracy of UA and FNA respectively is still not satisfied. Thus, it is very important to apply the combination of UA and FNA modalities for TNs diagnosis. Although numerous studies have reported to choose the combination of UA and FNA options for TNs diagnosis,^[21–23]^ no study has been conducted to investigate the diagnostic accuracy of UA and FNS for diagnosis of TNs.

## 2 Methods

### 2.1 Study registration

This study protocol has been registered on PROSPERO CRD42019138884. The reporting of this study is based on the guideline of Preferred Reporting Items for Systematic Reviews and Meta-Analysis (PRISMA) Protocol statement.^[24]^

### 2.2 Ethics and dissemination

No personal data will be used in this study, thus, this study will not need ethic approval. The results of this study will be published at peer-reviewed journals or conference proceedings.

### 2.3 Eligibility criteria

#### 2.3.1 Types of studies

We will include all case–controlled studies (CCSs) on the diagnostic accuracy of UA combined with FNA for TNs.

#### 2.3.2 Types of participants

We will include patients with histological proven TNs in this study.

#### 2.3.3 Type of index test

Index test: UA combined with FNA is used for TNs diagnose in the experimental group.

Reference test: Histological proven TNs are utilized for TNs diagnose in the control group.

#### 2.3.4 Types of outcome measurements

Sensitivity and specificity will be assessed as primary outcomes. Positive likelihood ratio, negative likelihood ratio, and diagnostic odds ratio will be evaluated as secondary outcomes.

### 2.4 Data sources and search strategy

#### 2.4.1 Electronic searches

We will adapt a comprehensive search strategy from the following electronic databases: MEDLINE, EMBASE, Cochrane Library, PsycINFO, Web of Science, Cumulative Index to Nursing and Allied Health Literature, Allied and Complementary Medicine Database, Chinese Biomedical Literature Database, and China National Knowledge Infrastructure. All databases will be searched from their inceptions to the present without language limitations. We will include all CCSs on investigating the impact of diagnosis UA combined FNA for TNs. The search strategy details for MEDILINE are showed in Table 1. Similar strategy will be applied to other electronic databases.

**Table 1:**
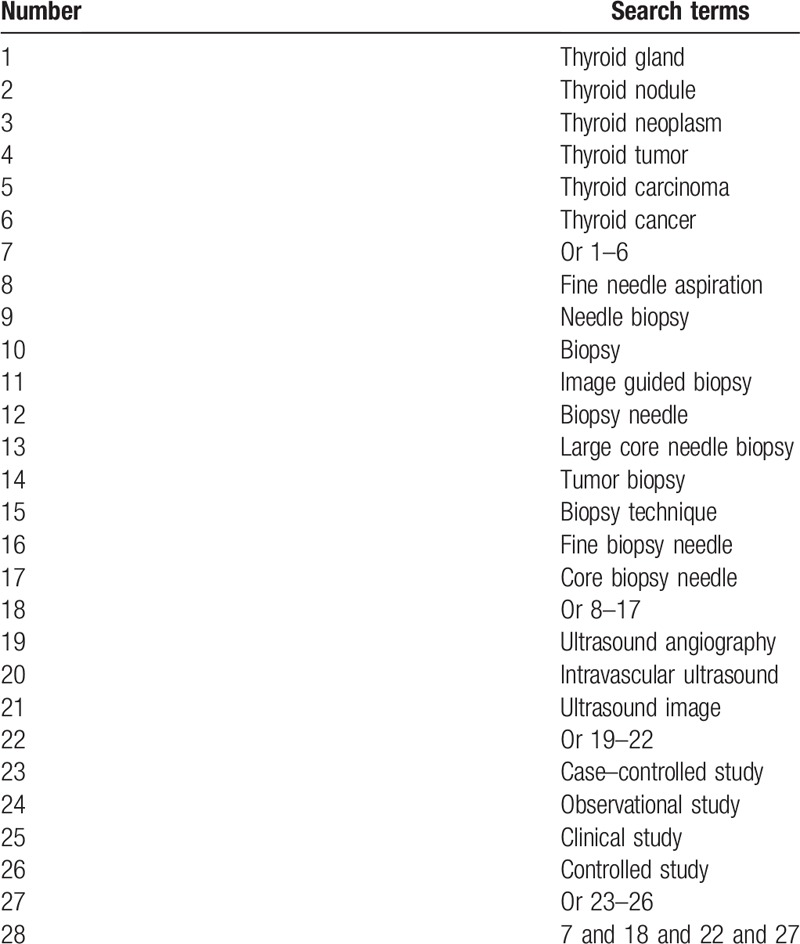
Search strategy for MEDLINE database.

#### 2.4.2 Other resources

We will also screen conference proceedings, and reference lists of included studies to obtain more potential studies.

### 2.5 Data collection and analysis

#### 2.5.1 Selection of studies

After comprehensive electronic search performance, the literature records will be managed by using EndNote X7 software (Philadelphia, USA). Two authors will independently carry out the study selection according to the previous designed eligibility criteria. Any disagreements will be solved by a third author through discussion. The whole process of study selection includes 2 stages. At first stage, the titles and abstracts of all literature records will be scanned, and all irrelevant records will be excluded. At the second stage, all remaining studies will be further reviewed by full-text, and each excluded study will be recorded with the specific reason. The study selection process is illustrated in the PRISMA diagram.

#### 2.5.2 Data collection and management

Two authors will independently extract the data for each included study. Any disagreements regarding the data extraction between 2 authors will be resolved by a third author through discussion. The items we will extract comprise of: basic characteristics (title, authors, publication year, region, journal, etc.), study design (study period, follow-up details, setting details, diagnosis of TNs, etc.), patient data (sample size in each group, tumor stage, comorbidities, etc.), and outcomes (measured tools, time points, results, etc.).

#### 2.5.3 Missing data dealing with

If there is insufficient or missing information, primary authors will be contacted to request those data. If those data are not achievable, only available data will be analyzed, and the potential effects of missing data will be evaluated and discussed.

### 2.6 Methodological quality evaluation

Two authors will independently assess methodological quality for all eligible studies using Quality Assessment of Diagnostic Accuracy Studies tool.^[25]^ It includes 4 domains, and each domain is further assessed. Any divergences regarding methodological assessment between 2 authors will be solved by a third author through discussion.

### 2.7 Statistical analysis

This study will apply RevMan V.5.3 (RevMan, London, UK) and Stata V12.0 softwares (College Station, USA) to perform statistical analysis. The descriptive statistics and 95% confidence intervals will be calculated for all included studies. In addition, descriptive forest plot and a summary receiver operating characteristic plot will be performed. The sensitivity, specificity, positive likelihood ratio, negative likelihood ratio, and diagnostic odds ratio from primary studies will be plotted.

#### 2.7.1 Assessment of heterogeneity

This study will apply *I*^2^ statistic to check heterogeneity for all included studies. The value of *I*^2^ ≤ 50% indicates low heterogeneity, while the value of *I*^2^ > 50% indicates substantial heterogeneity among eligible studies.

#### 2.7.2 Data synthesis

When the heterogeneity is low (*I*^2^ ≤ 50%), the data will be pooled, and meta-analysis will be carried out. When the heterogeneity is significant (*I*^2^ > 50%), the subgroup analysis will be performed, and meta-analysis will be carried out in accordance with the results of subgroup analysis. If the heterogeneity is still substantial after subgroup analysis, the outcome data will not be pooled, and meta-analysis will not be conducted. However, we will use bivariate random-effects regression to summarize the estimates of sensitivity and specificity.

#### 2.7.3 Subgroup analysis

Subgroup analysis will be carried out according to the different characteristics of studies, and participants.

#### 2.7.4 Sensitivity analysis

Sensitivity analysis will be performed to check the stability of pooled outcome results by eliminating the low methodological quality studies.

#### 2.7.5 Reporting bias

Reporting bias will be carried out by using funnel plots and relevant regression tests^[26]^ in this study.

## 3 Discussion

A variety of clinical studies have reported that the combination of UA and FNA is used for TNs diagnosis. However, its diagnostic accuracy is still inconclusive and no study has systematically explored the diagnostic accuracy of UA and FNS for TNs. Thus, this study will firstly investigate the diagnostic accuracy of UA and FNS in patients with TNs via evaluating its sensitivity, specificity, positive likelihood ratio, negative likelihood ratio, and diagnostic odds ratio. The findings of this study will summarize the latest evidence on the diagnostic accuracy of UA and FNS for TNs, and will provide helpful recommendation for the clinical findings of TNs.

## Author contributions

**Conceptualization:** Jing Wang, Xiao-Gang Bai, Zhe Liu.

**Data curation:** Jing Wang, Xiao-Gang Bai, Zhe Liu.

**Formal analysis:** Jing Wang, Xiao-Gang Bai.

**Funding acquisition:** Zhe Liu.

**Investigation:** Zhe Liu.

**Methodology:** Jing Wang, Xiao-Gang Bai.

**Project administration:** Zhe Liu.

**Resources:** Jing Wang, Xiao-Gang Bai.

**Software:** Jing Wang, Xiao-Gang Bai.

**Supervision:** Zhe Liu.

**Validation:** Jing Wang, Xiao-Gang Bai, Zhe Liu.

**Visualization:** Jing Wang, Xiao-Gang Bai, Zhe Liu.

**Writing – original draft:** Jing Wang, Xiao-Gang Bai, Zhe Liu.

**Writing – review & editing:** Jing Wang, Xiao-Gang Bai, Zhe Liu.
